# COVID-19 guidelines and media influenced ethical care in nursing homes

**DOI:** 10.1177/09697330241268923

**Published:** 2024-08-23

**Authors:** Caroline Wachtler, Monica Bergqvist, Pia Bastholm-Rahmner, Lars L Gustafsson, Katharina Schmidt-Mende

**Affiliations:** 27106 Karolinska Institutet; 27106 Karolinska Institutet; 27106 Karolinska Institutet; 27106 Karolinska Institutet

**Keywords:** COVID-19, ethical challenges, guidelines, media, nursing homes, qualitative study

## Abstract

**Background:**

The early phase of the COVID-19 pandemic affected nursing homes and their residents heavily. Guidelines on how to mitigate the virus’s spread and ensuring safe healthcare delivery were continually evolving. Concurrently, nursing homes faced intense media scrutiny. This challenging environment severely impacted registered nurses and physicians employed within these facilities.

**Aim:**

To understand the ethical challenges experienced by registered nurses and physicians working in nursing homes during the COVID-19 pandemic.

**Research design:**

Qualitative descriptive research using thematic analysis.

**Participants and research context:**

Individual online interviews with four registered nurses and eight physicians clinically active at nursing homes in Sweden.

**Ethical considerations:**

The study was approved by the Swedish Ethical Review Authority. All participants provided written consent.

**Results:**

Registered nurses and physicians working in nursing homes perceived ethical challenges stemming from early COVID-19 pandemic guidelines and media coverage. The main theme generated was ‘Struggling to maintain professional and ethical standards under pressure’ incorporating two subthemes: ‘Guidelines developed without the profession put pressure on staff’ and ‘Media’s biased reporting was perceived as unethical and undermined care’. Guidelines from the authorities were considered as developed without professional involvement. It made them difficult to adhere to without deviating from professional and ethical compasses. Media coverage adversely influenced relatives’ perceptions, resulting in mistrust towards physicians’ and registered nurses’ in delivering optimal care for the residents.

**Conclusions:**

Resilient care in nursing homes necessitates the collaborative development of guidelines involving registered nurses and physicians, particularly amidst crises. Moreover, it is vital to provide support to registered nurses navigating ethical dilemmas, especially during pandemics. Guidelines and principles for care during a crisis should be development with professional involvement, be transparent, and be available to the public, to promote neutral media coverage. Future research is crucial to enhance ethical standards and tackle challenges in this context.

## Introduction

During outbreaks of new diseases, ethically challenging scenarios arise. The early phase of the COVID-19 pandemic is such an example. Nursing home (NH) residents bore a disproportionate share of morbidity and mortality during the pandemic.^1^ Nearly half of all COVID-19 deaths took place in NHs.^2^ This demanding situation affected NH staff, many of whom experienced anxiety, stress, emotional exhaustion, moral and ethical dilemmas, feelings of abandonment, and disrespect.^3–6^ However, they also felt a sense of duty and commitment and were able to support each other.^5^ Additionally, NH healthcare staff faced unclear and constantly changing guidelines^7^ and perceived media criticism.^8^

Guidelines for NH care during the pandemic varied between and within countries and changed over time. COVID-19-related guidelines were often poorly conveyed, not well-suited to the context, and sometimes in conflict with healthcare workers ethical and professional values.^9–11^ In Scandinavia, these guidelines varied in extent and implementation. In Sweden, a recent policy analysis revealed that directives from authorities advising against physical visits to NHs had a detrimental effect on early pandemic care in these facilities.^9^ Box 1 describes the COVID-19 recommendations within the context of Swedish NHs.

**Box 1.** COVID-19 recommendation in the context of nursing homes in Stockholm, Sweden.

During the early COVID-19 crisis, outbreaks at NHs triggered media criticism about quality of care. Media portrayed NHs as dangerous for older people where the employees failed to maintain a professional care. This could be considered deceptive as it influenced public attitudes to NHs in a negative way and was not based on any scientific assessment of the professionalism of care provided.^4^ In Sweden, the wording used by the media depicted older people being especially vulnerable and in need of protection, while simultaneously portraying NH employees and facilities as hazardous for older people.^12^ A recent survey of front-line NH staff revealed negative media coverage as demoralizing.^3^

Considering the possibility of future pandemics or subsequent waves of COVID‐19, it is vital to address ethical issues that have been and remain prevalent. As healthcare adapts to the post-pandemic world, understanding how healthcare professionals responded to the initial COVID-19 crisis is critical to ensure future healthcare resilience.^13,14^ The aim of this study was to understand the ethical challenges experienced by registered nurses (RNs) and physicians working in NHs during the early COVID-19 pandemic.

• Approximately 108,000 people aged 65 years and older live in nursing homes (NHs) in Sweden. In May 2020, about 15.800 people in Region Stockholm (2.3 million inhabitants) lived in 318 NHs (median 76 residents, min 10, max 370).^15^
• NHs are tax-financed and the joint responsibility of the healthcare regions (physician service) and municipalities (registered nurses, nursing assistants, and management).
• Spring 2020: National COVID-19 care recommendations issued to prevent viral spread and control rate of hospital admissions.
• March 20, 2020 (revised April 1, 2020): Region Stockholm advises restricted hospital admissions for older people in NHs with Clinical Frailty Scale (CFS) score of 5 (living with mild frailty) or more and restricts NH on-site consultations.^16^
• June 2022: The National Board of Health and Welfare revises guidelines: physicians decide case by case if a physical visit is required.^17^

## Materials and methods

### Study design

In this study, we conducted reflexive thematic analysis^18^ of individual semi-structured interviews^19^ with physicians and RNs at NHs in Stockholm, Sweden. We followed the consolidated criteria for reporting qualitative research (COREQ)^20^ and were guided by Braun and Clarke’s checklist for quality practice in reflexive thematic analysis.^21^

### Setting and participants

We used the concept of information power to guide our estimation of sample size needed to answer the research question.^22^ Information power is a way of judging if the data collection will provide enough information to answer the research question and is seen as appropriate for studies using thematic analysis. Information power can be increased through five aspects of study design: when the study aim is narrow, when participants knowledge in the field is dense, when an established theory is used, when data have strong quality of dialogue, and when analysis is done across cases. Considering the focus of our research question, which delves into the experiences of a narrowly defined population within a specific context over a limited period, we aimed to recruit 8–12 participants.

As differences in burden of cases and socioeconomic locations might lead to different experiences, we recruited a purposive sample of RNs.^23^ We contacted managers at four NHs selected based on (1) diverse socioeconomic settings and (2) the proportion of COVID-19 deaths among NH residents. We asked managers to recruit one RN each. All RNs agreed to participate and were contacted by the research team for informed consent. We contacted physicians at each of these NHs by email and two agreed to be interviewed. Talking about the experience of working in NHs as a physician could be sensitive due to media criticism of care at NHs, which is why we continued recruitment using snowball sampling,^24^ starting with contacting physicians known to our research group and asking each participant to recommend individuals who might be interested in participation. We contacted a total of 36 physicians via either email or telephone, of which eight agreed to participate. Most non-participators did not give a reason for non-participation and those who did stated time limitations. In total we recruited twelve participants, four RNs and eight physicians, nine women and three men between 30 and 67 years of age. All were clinically active in NHs during the pandemic. We don’t have more specific information about their working conditions. During recruitment and data collection, we had weekly group meetings to discuss recruitment strategy and information power. Recruitment ended when the team felt the sample had adequate information power.^22^

### Data collection

We conducted individual semi-structured interviews between November 2020 and February 2021, using an online platform (Zoom^R^, Zoom Video Communications, San Jose, Ca). We informed participants before interviewing that our interest in conducting interviews was informed by our personal experiences of working with patients during the COVID-19 pandemic and our research interest to improve care for older frail individuals. In our interview guide we asked participants about their experiences of working in an NH during the pandemic, with four focus areas: (1) How work was affected, (2) support at workplace, (3) resources at workplace and, (4) cooperation at workplace and with other points of care. Informants were encouraged to talk about experiences from March 2020 to the date of the interview.

Participants were interviewed by one researcher. A second researcher observed and asked follow-up questions. Interviews were conducted by three of the authors and by three additional team members, all females. Four interviewers are currently clinically active in primary healthcare and worked clinically during the COVID-19 pandemic with older people receiving home healthcare. This meant we had shared experiences with our informants, although none of us had worked at an NH during the pandemic. Interviews lasted 50 to 75 min (median 60) and were audio recorded and transcribed verbatim in Swedish. We made transcripts anonymous to guarantee confidentiality.

### Data analysis

Our research group includes a behavioural scientist, a RN, a clinical pharmacologist, and three family physicians. Three of us are experienced qualitative researchers. We used a constructivist framework for the analysis with an understanding of knowledge as constructed through individual lived experiences and awareness that our lived experiences as researchers influence knowledge we generate in interaction with our research subjects and data.^25^ Interviews and analysis were conducted in Swedish. The analysis was completed manually.

First, all authors read the transcripts several times to grasp the entire material. The data set was organized by coding all text into meaningful units. Initially, the three qualitative researchers in our team separately coded and collated codes with similar content into potential subthemes. These authors discussed how potential subthemes were related by similar content or processes and generated potential themes. When opinions differed, we reread portions of the transcripts. Two of the researchers reviewed potential subthemes during repeated discussions and merged subthemes considered to have a common origin, constructed refined themes, and identified illustrative quotes in the data. The third qualitative researcher conducted reciprocal reading between transcripts and themes/subthemes to ensure no data was overlooked, to further refine the name and definition of each theme, and to identify illustrative quotes. At this point, all quotes were translated from Swedish to English by one of our team (a native English speaker). All authors iteratively reviewed the text before final approval.

### Ethical considerations

Participants received written and oral information about the study aim and how results would be used. They were informed that participation was voluntary, that they could withdraw at any time without providing a reason, and that data were treated confidentially. All participants provided written consent. The Swedish Ethical Review Authority approved the study (Dnr 2020-03720).

## Findings

**Figure 1.**
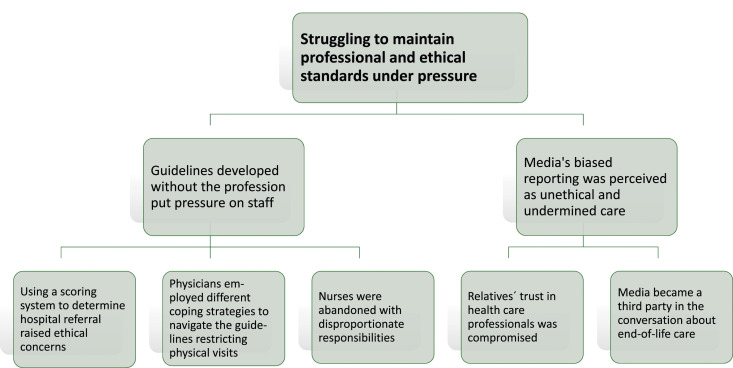
Themes and subthemes outlining the challenges experienced by registered nurses and physicians in nursing homes due to guidelines and media attention.

We generated one major theme from interview data: ‘Struggling to maintain professional and ethical standards under pressure’. The central concept in the interviews was that professional and ethical standards were threatened during the pandemic. Respondents reported that fear of infection, lack of adequate personal protective equipment (PPE), and the individual suffering and loneliness of the residents all put pressure on them during the pandemic. For these respondents, however, the emphasis of the content of the interviews was on feeling hindered by external pressures in their ability to work according to professional and ethical standards. These perceived external pressures are described in two subthemes: (1) ‘Guidelines developed without the profession put pressure on staff’ based on the relationship between three categories of data and (2) ‘Media’s biased reporting was perceived as unethical and undermined care’ based on two categories of data (Figure 1).

### Guidelines developed without the profession put pressure on staff

Participants described how the guidelines aimed at reducing hospitalizations through the use of the Clinical Frailty Scale (CFS) and the restriction of physician visits to NH residents negatively affected their work. RNs and physicians felt that guidelines forced them to make decisions that were not in line with their professional ethics. Respondents were frustrated and felt that their expertise had been overlooked when the guidelines were developed and that those who developed the guidelines had no clinical experience from NHs. Participants describe that using a scoring system to determine hospital referral raised ethical concerns. Physicians employed different coping strategies to navigate the guidelines restricting physical visits.

#### Using a scoring system to determine hospital referral raised ethical concerns

The RNs and the physicians agreed that using the CFS to assess frail persons for hospital referral instead of making an individual assessment was not in line with the ethical and professional compass they usually rely on. According to the guidelines, only individuals with a score less than five were to be referred to the hospital, implying no NH resident should be sent to the hospital for medical care as most NH residents have a CFS score of five or above.

I’m not sure we would have sent that many more to the hospital during the first wave if we didn’t have that document. But it would have saved us a lot of discussion and criticism. I think, trust us more who work at the nursing homes, we have some idea about what we are doing (Physician No. 3).

#### Physicians employed different coping strategies to navigate the guidelines restricting physical visits

**Figure 2.**
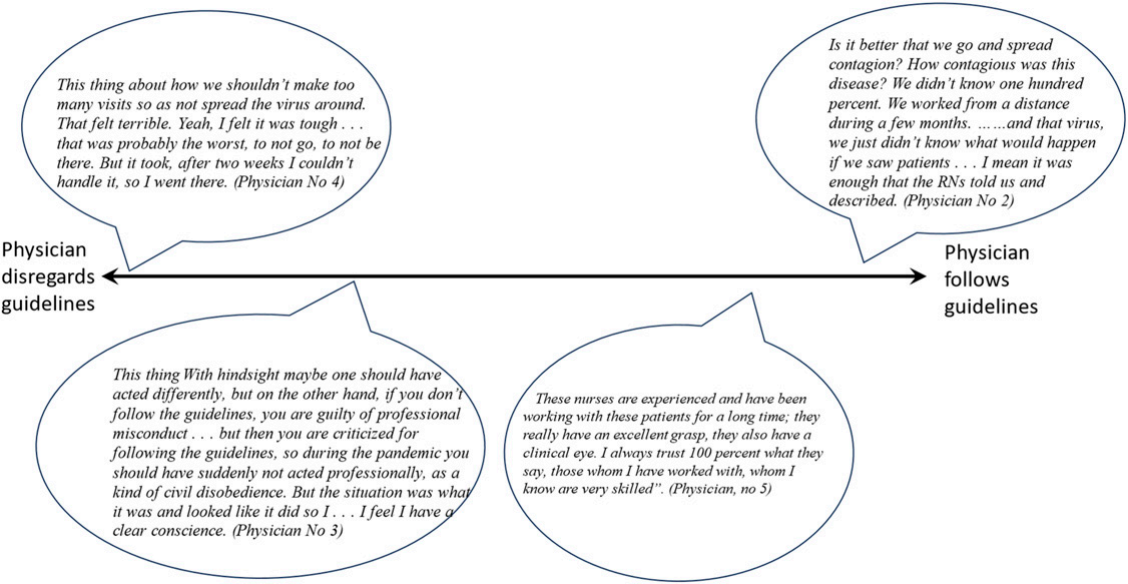
Spectrum of physicians approaches to guidelines restricting physical visits.

Guidelines restricting physical visits to the NHs challenged physicians’ ability to make adequate medical decisions and to provide support for the RNs. Physicians adopted a spectrum of strategies for adhering to these guidelines to be consistent with their individual medical and ethical standards (Figure 2). One strategy was to defy the guidelines and rely on their own ethical judgements and compass. Another strategy was to accept that there was an inherent conflict between following guidelines and supporting residents and RNs. This strategy allowed physicians a clear conscience regarding guidelines but could result in dissatisfaction and unsurety about the decision. Some physicians followed guidelines without exceptions, expressing that avoiding contagion was prioritized over physical visits to the NHs. While physicians using this strategy recognized that staff might feel abandoned, they pointed out that as physicians they are not part of the NH team and have no responsibility for how the NH is run. Additionally, some physicians believed that the nurses possessed sufficient knowledge to assume responsibility for medical care, with the physician serving as a consultant at distance.

#### Nurses were abandoned with disproportionate responsibilities

Under normal circumstances, each NH has at least one manager present during the day and a physician who visits weekly or as needed for resident care. To mitigate the spread of the virus, Sweden’s Public Health Agency encouraged those capable of working from home to do so. As a result, in some NHs, both managers and physicians primarily conducted their work from a distance. RNs expressed they were put in the line of fire to perform good care and to make difficult decisions while both physicians and managers were physically absent from the NHs due to recommendations in the guidelines. RNs felt overwhelmed by disproportionate responsibilities.

I remember standing there in the doorway just looking at him (the resident) seeing the panic in his eyes (participant crying) . . . and I just . . . what are we doing? I can honestly say I did the best I could to make sure they were ok, but I do not feel they received quality care (Registered Nurse No. 1).

RNs reported they had to take medical decisions for severely ill patients alone. They informed physicians about residents’ symptoms and signs over the telephone. Some RNs felt forced to conduct tasks that in general are a physician’s responsibility such as end-of-life dialogues with the residents and their relatives or decisions on when to initiate palliative care.

The physician trusts what we say . . . so he wasn’t here with us making clinical examinations but instead based his decisions about end-of-life care on us. And I think it is just so wrong (participant crying) because I don’t have that training, I am not a doctor, and I don’t have that responsibility either (Registered Nurse No. 2).

RNs felt obliged to develop and maintain new routines for nursing assistants who often lacked basic hygiene knowledge as there were no managers in place and, in the absence of established, sustainable hygiene guidelines.

I had to sort of pretend I knew what I was doing. I had to make decisions that were far above my level. And I had to pretend I knew what I was doing to not spread panic. And I myself had a little panic because I didn’t know anything (Registered Nurse No. 3).

### Media’s biased reporting was perceived as unethical and undermined care

Physicians and RNs experienced that media coverage accused healthcare professionals of spreading contagion to residents and circulating misleading information about palliative care. As a result, relatives’ trust in healthcare professionals was compromised and the media emerged as a third party in conversations, undermining professional and ethical standards for end-of-life care. The perceived negative and inaccurate reporting by the media resulted in relatives´ loss of trust in physicians and RNs. Participants described experiencing a dual challenge: on one hand, their expertise was scrutinized by media coverage; on the other hand, they still had to navigate the constraints of restrictive guidelines.

#### Relatives’ trust in healthcare professionals was compromised

Media coverage picked up the high death rates in the NHs, coupled with reduced referrals to hospital and few physical visits by physicians and began to question the way NHs provided care. While RNs and physicians aimed to increase or retain the residents’ quality of life, media focused on survival at any cost. Participants wished that media provide a more nuanced picture about care at NH and wished they had been given the opportunity to make a statement in the media themselves as they had experience and knowledge about this group of frail older people.

I think it was very accusing of us in elderly care, and there was misuse of the term palliation – that it was about killing people rather than being about easing symptoms at the end of life. They think oh help now my relative is going to die suffering alone in the room. And that is the impression they get in the media, that they will die alone, and they will die in suffering, that we do nothing at the nursing home (Registered nurse No. 3).

#### Media became a third party in the conversation about end-of-life care

Media became a third party in conversation as unbalanced descriptions of palliative care caused mistrust and fear among relatives. As a consequence, physicians found it hard to make decisions about palliative care. This gave rise to complicated conversations and many long phone calls with residents’ relatives where physicians had to explain and often defend their medical decisions.

Parallel to all this the debate in the media started which was very stressful, I slept badly, there were so many more contacts and long discussions with relatives who had heard that we practiced active euthanasia. We were called murderers in the comments on social media and so on (Physician No. 5).

Relatives could insist on hospital referral despite the physician considering that a referral would not improve the resident´s chances of survival but instead entail increased suffering. Physicians were sometimes hesitant to initiate palliative care. It happened that they referred residents to hospital out of fear of being criticized by relatives, even if they considered that referral was medically inappropriate. Physicians felt undermined in their ability to take medically informed, person-centred decisions.

But what we normally are very good at is this that as geriatricians we should think about yeah, that life might not be that much longer now and how do we think about that. Planning for death with dignity. That is really our specialty. But it has become uncomfortable. We are used to having those discussions. But now it feels we don’t really dare because it can raise irritation and fear (Physician No. 8).

## Discussion

We found that RNs and physicians working in NHs in Sweden faced ethical challenges arising from early COVID-19 pandemic guidelines and media’s coverage. The authorities’ guidelines limited and pressured professionals in their ability to meet professional and ethical standards and were seen as not having been collaboratively developed with the professionals who have expertise in the care of frail older people. Additionally, media coverage adversely influenced the perceptions of relatives, resulting in diminished trust in physicians and RNs and undermining professionals in their goal to provide optimal care for NH residents. Collectively, these factors caused ethical and moral distress among RNs and physicians. Healthcare professionals in NHs regularly face and navigate ethical challenges in caring for old, frail persons near the end of life.^26^ As witnessed by our participants, as well as in other research, these challenges increase during a crisis.^27,28^

Both RNs and physicians in our study were pressured to accommodate guidelines restricting hospital referrals by using the Clinical Frailty Scale (CFS) score for decision-making. This left RNs and physicians in conflict with their ethical principles and knowledge on one side and pressure to adhere to guidelines on the other as they had to balance individual needs with collective demands. In many countries, triage recommendations during COVID-19 have been seen as controversial and caused debates.^29^ In the UK, healthcare workers described how pandemic guidelines were in tension with professional values, did not accommodate contextual considerations, were inconsistently communicated,^10^ or simply developed without scientific rigour.^30^ Similarly, critical voices in Sweden have highlighted that while the CFS estimate may offer a broad framework for resource allocation at group level, it should not be utilized to determine the prioritization of care interventions for individual patients.^31–34^

The guidelines restricting physical visits to the NHs for physicians were not in line with most participants’ professional and ethical compass. It is well known that the implementation of guidelines in the complex NH setting is demanding.^35^ In a Norwegian study, pandemic guidelines were sent to healthcare workers for comments and thereafter substantially revised, which may explain why healthcare staff widely accepted those recommendations.^36^ Physicians in the current study varied considerably regarding how they handled the guidelines which in turn affected the RNs in different ways. One strategy was to defy the guideline restricting physical visits and to refuse to work remotely, while other physicians strictly followed the guidelines and stayed at a distance. This may be partially explained by how physicians experienced their autonomy and role in the NH team. While some of the physicians considered themselves part of a team, others saw themselves as a consultant and not part of the NH team.

Research in the intensive care setting has shown that restrictions on clinical autonomy can lead to moral distress, especially for more experienced physicians,^37^ and it is possible that there is a similar relationship for physicians in this study. Previous research indicates that sharing views and discussing ethical dilemmas, both during ordinary circumstances and during crisis situations, can mitigate differences in opinions and help maintain high-quality care guided by respect and ethical integrity at NHs.^38^ Professional discussions about ethical dilemmas and support from managers can mitigate the consequences of crisis including increased job satisfaction, affective commitment, and prevention of staff burnout.^39^ A Norwegian study highlighted the profound impact of the pandemic on physicians working at NHs, presenting them with intricate ethical dilemmas of which the primary concern was balancing the well-being of individual residents with the needs of the larger community.^40^ Similarly, our analysis shows that physicians reported ethical challenges due to the pressure of accommodating guidelines. Furthermore, to frame the concept of moral stress is complex and currently defined in different ways in different disciplines,^41,42^ and the concept needs to be further developed in order to create common ground for development of preventive measures for the negative consequences of this phenomenon. Gustavsson et al. have developed a new definition that may be useful: ‘Moral stress is the type of stress that occurs when one is confronted with a moral challenge, a situation in which it is difficult to resolve a moral problem and in which it is difficult to act, or to feel inadequate when acting, in accordance with one’s own moral values’.^11^ Another way to develop the concept of moral stress and better understand its consequences would be to interview RNs and physicians about situations where they have experienced moral stress and how it manifested for individual subjects. This would provide a base to design preventative measures for the negative consequences of the moral stress.

The RNs in this study described feeling abandoned with sparse support from physicians or managers as they struggled to provide quality care. They expressed increased responsibility for end-of-life communication with relatives, and for assessing deteriorating health conditions of residents, giving descriptions over the phone to the physician while also taking responsibility for staff compliance with hygiene routines. Similarly, a study conducted in Germany during the pandemic showed RNs in NHs had to take on increased medical responsibilities such as ordering or discontinuing medication or actively deciding on medical treatment in critical situations.^43^ A Norwegian study reported that due to limited staff availability, a single NH nurse could be the only staff member who knew the residents, was competent to perform the hygiene measures, and knew the ward procedures. These nurses expressed how they were overwhelmed by responsibilities and felt unable to practice soundly.^7^

Physicians’ and RNs decision-making regarding palliative care was affected by media reporting, leading relatives to mistrust physicians’ assessments and decisions. Negative and inaccurate reporting by the media resulted in relatives no longer trusting physicians and RNs. It is well known that negative media reports can influence public perceptions and behaviours.^3,8^ The ways the media present news influences how the public perceives provision of care in NHs.^4^ Healthcare workers in Sweden and worldwide have felt demoralized by negative reports in traditional and social media stating that healthcare workers transmitted the virus to residents.^8,44^ Previous research indicated that media reports have had detrimental effects on managers, their staff, and the overall well-being of NH residents.^37^ NH managers in Sweden reported that they found media coverage of their work during COVID-19 harsh, negative, and incomplete.^3^ In that study, as well as in the current study, participants expressed frustration over the lack of understanding about the role of NHs, in particular misunderstanding around end-of-life care. At the same time, negative media coverage brought attention to the insufficient number of educated staff and resources, leading to political measures in Sweden to address these deficiencies and enhance the allocation of resources to NHs.^32,45,46^

Our study shows that clinical decision-making was negatively affected by both guidelines and media reporting. Similar results were found in a Norwegian study suggesting that both national and local guidelines constrained RNs professional decision-making because the guidelines were developed to maximize overall societal utility rather than catering to the needs of individual frail residents.^7^ A Swedish study in a home healthcare setting showed different results. The physicians responded to the pandemic crisis by taking explicit responsibility for prioritizing and resolving ethically challenging situations. In the context of increased risk of death, physicians were able to cultivate the patient perspective in end-of-life discussions and to adapt care to the individual.^14^ The difference between this and the current study may be that different guidelines apply to home healthcare than to NH.

Guidelines for care during a crisis/pandemic should always address the ethical principles and practice for NHS to ensure resilient care. A key principle for effective implementation is the need that NH guidelines and principles for care during a crisis should be developed and implemented with RNs and physicians who will use them. Triage principles at NHs should be presented, founded on individual clinical assessments of needs and best possibilities for cure care and nursing. These principles in the public health model should be part of education programmes for all healthcare and nursing staff and made publically known. Clarity in guidelines and nursing planning can promote trust of the public and media on the quality of nursing and care of the most fragile old during a pandemic/crisis, and can help ensure that resources are provided to NHs as well as to emergency care.

## Strength and limitations

This study has some methodological limitations. RNs were recruited through NH managers, who may have selected RNs with biased experiences and views. However, we found that participants freely expressed criticism about how care was given during the pandemic. The study could have been strengthened by further recruitment of RN participants, and we may have missed some important perspectives. However, the four RNs in the study worked in different NH environments during the pandemic but yet reported similar experiences with high information power. Physicians were difficult to recruit, perhaps because of widespread negative media reporting. We therefore used snowball sampling to recruit physicians, a useful strategy for investigating problematic situations, and while it may limit the kinds of experiences captured, snowball sampling allows access to groups that would otherwise not be heard from at all.^24^ Interviews were strengthened by joint participation of an interviewer and an observer, and by the interviewers also being healthcare professionals who had worked during the early pandemic. The digital format allowed participants to choose the interview environment. Thematic analysis of individual interviews is appropriate when aiming to find a pattern of meaning across a data set, as in our study.^18^ Information power is preferable to data saturation as an approximation of content validity in thematic analysis.^22^ This study had a relatively narrow aim, high sample specificity, data rich in content with detailed descriptions, and a cross-case analysis strategy, meaning our sample size was adequate to provide information power.^22^ The analysis was enriched by multiple researchers with different healthcare backgrounds and by multiple coders with different experiences who discussed similarities and differences in the coding process as part of theme generation. It is possible that the analysis would have been further enriched by participation of researchers without healthcare backgrounds, by repeated interviews, or by involving participants in the analysis process, but these were beyond the scope of the project. Transparent reporting of methodological processes allows readers to identify transferability of our findings in other clinical settings.

## Conclusion

Guidelines ensuring resilient care in NH guidelines should be developed and implemented with RNs and physicians who will use them, especially during a crisis. It is important that RNs are supported by managers and physicians in ethically challenging situations such as a pandemic. Discussions about media´s ethical responsibility to report in a balanced way have to continue. Further research is necessary to promote the establishment of common ethical standards and facilitate discussions about ethical dilemmas that arise for nurses and physicians in NHs. This involves a concerted effort to refine guidelines that both professions can follow. By promoting a collaborative approach to ethical considerations, healthcare professionals can better navigate complex situations and maintain the highest standards of resident care. In addition, such studies can form the basis for improving education and ensuring that RNs and physicians are equipped with the knowledge and skills necessary to deal with ethical challenges. Ultimately, the development of common ethical frameworks will benefit the well-being and quality of life of residents as well as healthcare workers.
